# COVID-19 Testing, Vaccine Perceptions, and Trust among Hispanics Residing in an Underserved Community

**DOI:** 10.3390/ijerph20065076

**Published:** 2023-03-14

**Authors:** Gabriel A. Frietze, Bibiana M. Mancera, Michael J. Kenney

**Affiliations:** Border Biomedical Research Center, College of Science, University of Texas at El Paso, 500 W. University Ave., El Paso, TX 79968, USA

**Keywords:** COVID-19, Hispanics, trust, vaccine uptake, perceived effectiveness

## Abstract

The Borderplex region has been profoundly impacted by the COVID-19 pandemic. Borderplex residents live in low socioeconomic (SES) neighborhoods and lack access to COVID-19 testing. The purpose of this study was two-fold: first, to implement a COVID-19 testing program in the Borderplex region to increase the number of residents tested for COVID-19, and second, to administer a community survey to identify trusted sources of COVID-19 information and factors associated with COVID-19 vaccine uptake. A total of 4071 community members were tested for COVID-19, and 502 participants completed the survey. COVID-19 testing resulted in 66.8% (*n* = 2718) positive cases. The community survey revealed that the most trusted sources of COVID-19 information were doctors or health care providers (67.7%), government websites (e.g., CDC, FDA, etc.) (41.8%), and the World Health Organization (37.8%). Logistic regression models revealed several statistically significant predictors of COVID-19 vaccine uptake such as having a trusted doctor or health care provider, perceiving the COVID-19 vaccine to be effective, and perceiving that the COVID-19 vaccine does not cause side-effects. Findings from the current study highlight the need for utilizing an integrated, multifactorial approach to increase COVID-19 testing and to identify factors associated with COVID-19 vaccine uptake in underserved communities.

## 1. Introduction

The novel SARS-CoV-2 virus was first detected in January 2020 in Hubei Province, China and led to the global coronavirus pandemic [1]. The Unites States confirmed its first COVID-19 case on 20 January 2020, and on 13 March 2020, El Paso, Texas reported its first COVID-19 case [2]. The SARS-CoV-2 pandemic exposed many established causal relationships between co-morbidities, social determinants of health, marginalization, and inequities among people of color, particularly Hispanics. Hispanics in the U.S. are more than three times as likely to become infected by the SARS-CoV-2 virus and age-adjusted hospitalization rates are 4.6 times higher when compared to non-Hispanic whites (NHWs) [3,4,5,6,7]. There are multiple factors that likely contribute to high COVID-19 infection rates and negative health outcomes among Hispanics, including social determinants of health inequities. While Hispanics comprise 18.9% of the entire U.S. population, within the county of El Paso, Texas, Hispanics account for nearly 83% of the population [8,9]. El Paso is part of a unique region known as the binational Borderplex, which includes southern Doña Ana County, New Mexico and Ciudad Juarez, Chihuahua, Mexico. Together, their combined populations exceed 2.5 million people. Although a state line and an international border separates these states, a fluid community exists that commutes daily for work, family, and healthcare.

The Borderplex region has been profoundly impacted by the SARS-CoV-2 pandemic. In El Paso alone, over 301,912 people have reported a COVID-19 infection, and there have been over 3626 deaths [10]. This reality exposed stark differences in underserved communities within the Borderplex region related to access to healthcare, COVID-19 testing, and health statuses. Underserved communities can be described as areas that lack sufficient access to healthcare and include rural, blue collar, low-literacy, and elderly populations [11]. Moreover, underserved groups include minority populations such as Hispanics, African Americans, and refugees, who experience health disparities due to economics, cultural and language barriers, transportation, and a limited number of healthcare providers [11]. Many highly vulnerable Borderplex residents were under-tested during the SARS-CoV-2 pandemic, a significant omission as these individuals likely have underlying medical conditions such as obesity, diabetes, and cardiovascular disease. Additionally, many households within the Borderplex are multigenerational and household members are “essential workers” in low-paying frontline jobs. Low socioeconomic status (SES) neighborhoods within the region lacked substantive COVID-19 testing and contributed to the “testing deserts”. COVID-19 testing programs in the Borderplex region are critical for increasing access to testing and reducing spread in underserved communities.

To increase access to COVID-19 testing in the Borderplex region, the Border Biomedical Research Center (BBRC) at the University of Texas at El Paso (UTEP) was funded in the fall of 2020 by the National Institutes of Health (NIH) RADX-UP program (grant # 3U54MD007592-27S1), a supplement associated with the parent NIH U54 previously awarded to UTEP (grant # 5U54MD007592-27). The funded project aimed to: (1) reduce testing deficiencies by providing education, SARS-CoV-2 testing information, and navigation strategies to testing sites and (2) to identify the factors associated with COVID-19 vaccine uptake including trusted sources of COVID-19 information (e.g., the U.S. government) and COVID-19 health perceptions (e.g., fear of COVID-19 vaccine side effects). We capitalized on the demographic advantages of the El Paso region, the extensive expertise of an interdisciplinary investigative team, the core facilities, the analytical capabilities of the BBRC, and the established collaborations between UTEP, local hospitals, the City of El Paso Department of Public Health, and regional nonprofits and businesses to enhance multidimensional testing and research initiatives.

In addition to increasing COVID-19 testing, we sought to examine the sentiments toward COVID-19 vaccines in residents of the Borderplex region. Recent studies examining public opinions about COVID-19 vaccines have used social media platforms to investigate vaccine discussions [12,13,14]. For example, “tweets” from public profiles on Twitter have been used as qualitative data to examine COVID-19 vaccine opinions. Several important findings have emerged using qualitative data from Twitter, suggesting that machine learning can help detect COVID-19 vaccine misinformation on Twitter [12], multilayer network-based approaches can be used to understand characteristics from “pro-vaxxers”, “anti-vaxxers”, and “neutral” individuals who post on Twitter [13], and sentiments about COVID-19 vaccines that are posted to Twitter can be coded into various categories such as positive, negative, or neutral to understand vaccine opinions from millions of Twitter users [14]. Findings from these studies have implications for developing health messages that are effective at reducing vaccine hesitancy and increasing vaccine acceptance in the general public, without consideration of ethnicity.

Given that COVID-19 health outcomes are worse for Hispanics than White Americans, research investigating COVID-19 vaccine hesitancy and acceptance in Hispanics is warranted. A recent review examining COVID-19 vaccine hesitancy among Black, Hispanic, and undocumented immigrants reported that attention towards vaccine hesitancy has focused more on Black communities than Hispanic communities [15]. Specifically, the authors report that their literature search using PUBMED yielded 10 articles that included results for Hispanic Americans compared to 50 studies that focused on Black Americans [15]. Moreover, of the 10 studies that included results for Hispanic Americans, none focused exclusively on Hispanics [15]. Investigating differences in ethnicity has contributed to understanding key factors that contribute to vaccine hesitancy and vaccine acceptance, including factors such as medical distrust from ongoing or historical medical mistreatment [16]. A qualitative study of Hispanic and Black individuals in New York suggests that having a trusted doctor who recommends the COVID-19 vaccine is a key driver for COVID-19 vaccine acceptance [17]. There is a need for further research using a quantitative approach to examine factors associated with COVID-19 vaccine acceptance in Hispanics. The current study addresses this gap.

We sought to answer three research questions. The first question we addressed was whether we could increase COVID-19 testing in the Borderplex region through the UTEP Coronavirus Testing Program. The second research question was to identify who individuals trust for COVID-19 information. The final research question was to identify what factors are associated with COVID-19 vaccine uptake in individuals residing in the Borderplex region. This study is among the first to develop a program to increase COVID-19 testing and concurrently assess perceptions about COVID-19 in an underserved predominately Hispanic population.

## 2. Materials and Methods

### 2.1. Participants

#### 2.1.1. COVID-19 Testing in Borderplex Residents

A total of 4071 community members were tested from 1 January 2022 to 8 July 2022. More than half of the 4071 community members tested positive for COVID-19 (*n* = 2718), and their positive results were examined for further analysis. Nasopharyngeal swabs were utilized by trained collection personnel to obtain samples from testing participants. Community health workers (CHWs) were deployed to navigate community members to testing sites. CHWs were particularly useful for navigating community members who were not fluent in English and/or of lower SES.

#### 2.1.2. Community Survey Administered to Borderplex Residents

Five hundred and two adults (*M*_age_ = 33.79, *SD* = 9.15; female = 52.4%, *n* = 263) who were predominately Hispanic (66.7%, *n* = 335) participated in the community survey. To promote non-physical contact, Facebook geo-targeted advertisements were used to recruit and retain eligible participants. Only participants 18 years of age and older and who reported residing in the borderland region (El Paso, New Mexico) were eligible to complete the survey.

### 2.2. COVID-19 Testing Measures

The COVID-19 test administered to community members was a nucleic acid/PCR test. The MagMAX Vira/Apthogen Nucleic Acid Isolation kit (Thermo Fisher, Waltham, MA, USA) and the KingFisher Flex Purification System (Thermo Fisher, Waltham, MA, USA) were utilized and viral SARS-CoV-2 RNA was confirmed via multiplex quantitative real-time PCR (qRT-PCR) adhering to the Applied Bioscience TaqPath COVID-19 Combo Kit. In addition, cDNA from viral RNA was produced using the Invitrogen’s SuperScript IV First-Strand Synthesis System. This was followed by a library construction from the first strand cDNA utilizing IDT’s xGen SARS-CoV-2 for SARS-CoV-2 containing primers against the complete genome SARS-CoV-2 isolate Wuhan-Hu-1 (NCBI Reference Sequence NC_045512.2).

### 2.3. Community Survey Measures

A cross-sectional survey was designed for an inventory assessment of COVID-19 related factors (e.g., prevention, testing, and future vaccine acceptability) among a Hispanic majority sample. Items included within the survey were adapted from the literature, created by the authors, or provided by the Rapid Acceleration of Diagnostics-Underserved Populations (RADx-UP) common data elements managed by Duke University. The RADx-UP common data elements assess a variety of factors related to demographics, health information, and COVID-19-related information.

#### 2.3.1. Trust in Sources of Information

Several items assessed who individuals trust for COVID-19 information. Sample item: “How much do you trust each of these sources to provide correct information about COVID 19?” Eight response options were provided: (1) your faith leader, (2) your doctor or health care provider, (3) your close friends and members of your family, (4) people you go to work or class with or other people you know, (5) news on the radio or tv, online, or in newspapers, (6) your contacts on social media, (7) the U.S. government, and 8) the U.S. Coronavirus Task Force. The eight items were rated on a 4-point scale ranging from (1) not at all to (4) a great deal. This item was included from the RADx-UP common data elements described above.

An additional item explored trustworthy messengers of COVID-19 information: “If you were receiving information about COVID-19 and the COVID-19 vaccine, who would you believe is the most credible? (Select all that apply)”. Nineteen response options were provided: “Healthcare practitioner (e.g., pediatrician, family practice doctor)”, “communication health clinic”, “Pharmacy (e.g., Walgreens, CVS, etc.)”, “Pharmacist”, “school nurse”, “family/friends”, “social worker”, “social media”, “Government website (e.g., CDC, FDA, etc.)”, “World Health Organization (WHO)”, “television”, “radio”, “internet”, “newspaper”, “Community health worker or health promotor(s)”, “elected officials”, “public health official”, “university communications”, and an “other” option which permitted text entry. Responses were coded as 1 = yes and 0 = no. Participants were given the opportunity to select more than one response option. The latter item was created by the authors of the current study.

#### 2.3.2. Perceived Efficacy of COVID-19 Protective Behaviors

Five-items from Clark et al. (2020) assessed assumptions regarding the efficacy of behavioral health precautions (α = 0.72) [18]. Sample items: “Avoiding crowds is an effective method for fighting COVID-19”, “Practicing social distancing is an effective method for avoiding COVID-19”, “Staying at home is an effective method for avoiding COVID-19”, “Washing your hands frequently is an effective method for avoiding COVID-19”, and “Wearing a surgical mask is an effective method for avoiding COVID-19.” Response options ranged from (1) strongly disagree to (5) strongly agree. A composite was created by averaging the five items. The Cronbach alpha for the five items was equal to 0.91 in the current study.

#### 2.3.3. Perceived COVID-19 Invulnerability

Five-items from Clark et al. (2020) assessed perceived invulnerability (α = 0.68) [18]. Sample items: “I am less likely than most people to get COVID-19”, “I am not at risk for getting infected with COVID-19”, “My body could fight off COVID-19 infection”, “People like me don’t get COVID-19”, and “There is little chance that I could get or spread COVID-19 from what I do in my everyday life”. Response options ranged from (1) strongly disagree, to (5) strongly agree. A composite was created by averaging the five items. Lower scores indicate that participants feel more susceptible to COVID-19. The Cronbach alpha for the five items was equal to 0.83 in the current study.

#### 2.3.4. Perceived Effectiveness of the COVID-19 Vaccine

Two items created by the authors assessed perceived effectiveness of the COVID-19 vaccine at preventing infection and sickness: “I believe the COVID-19 vaccine will be effective in preventing the infection” and “I believe if I get the COVID-19 vaccine, I will be less likely to get sick.” Response options ranged from (1) strongly disagree to (5) strongly agree. A composite was created by averaging the two items. The correlation between the two items was equal to 0.72 in the current study.

#### 2.3.5. Fear of COVID-19 Vaccine Side-Effects

Three items adapted from Brabin et al. (2006) assessed fear of COVID-19 vaccine side-effects: “I worry that the COVID-19 vaccine might negatively affect me”, “I worry about the short-term side effects of the COVID-19 vaccine”, and “I worry that the COVID-19 vaccine might have unknown long-term side effects” [19]. Response options ranged from (1) strongly disagree to (5) strongly agree. A composite was created by averaging the three items. The Cronbach alpha for the two items was equal to 0.86 in the current study.

#### 2.3.6. Personal Health Importance

Three-items from Clark et al. (2020) assessed personal health importance (α = 0.74). Sample items: “I am concerned about my health and am taking action to prevent COVID-19”, “My health is my top priority”, and “Taking care of my health means a lot to me” [18]. Response options ranged from (1) strongly disagree to (5) strongly agree. A composite was created by averaging the three items. The Cronbach alpha for the three items was equal to 0.78 in the current study.

#### 2.3.7. COVID-19 Vaccine Uptake

A single item assessed if participants have received the COVID-19 vaccine. Sample item: “Have you received a COVID-19 vaccine?” Response options included: 0 = No, 1 = Yes, 98 = Prefer not to answer.

### 2.4. Community Survey Procedure

Participants completed the 30–45 min online survey using personally owned computers, tablets, or cell phones. The survey was created using QuestionPro survey software (Questionpro, Austin, TX, USA). A $30 electronic gift certificate was provided to participants upon completing the survey as compensation. Our study was approved by our universities IRB and all guidelines for human subjects were followed for recruitment and participation of subjects.

### 2.5. Approach to Analysis

Descriptive statistics were calculated for basic demographic and background information. Two logistic regression models examined factors associated with COVID-19 vaccine uptake (yes/no). Model 1 examined trusted sources of COVID-19 information. Specifically, COVID-19 vaccine uptake was entered as the dependent variable, and eight independent variables were included in the order they were assessed in the original survey (faith leader, doctor or health care provider, close friends and members of your family, people you go to work or class with or other people you know, news on the radio/TV/online/newspapers, contacts on social media, the U.S. government, and the U.S. Coronavirus Task Force). Model 2 examined COVID-19 health perceptions. Specifically, COVID-19 vaccine uptake was entered as the dependent variable, and five independent variables were included (perceived efficacy of COVID-19 protective behaviors, perceived COVID-19 invulnerability, perceived effectiveness of the COVID-19 vaccine, fear of COVID-19 vaccine side-effects, and personal health importance).

## 3. Results

### 3.1. COVID-19 Testing Results

A total of 2718 community members tested positive for COVID-19. Approximately half (52.8%; *n* = 1392) of the positive cases were tested at a drive-through testing site through the city, and 48.7% (*n* = 1325) were tested in a lab setting (University Medical Center).

### 3.2. Community Survey Results

Approximately 42.8% of respondents reported an education below a bachelor’s degree and half of the sample (50.2%) reported a household annual income less than $50,000. See Table 1 for participant demographic characteristics. A third (33.9%) of our sample identified as essential workers. Nearly a fifth (18.9%) of our sample reported having no health insurance, and 22.5% reported that they had lost their health insurance due to the COVID-19 pandemic. Most of the sample reported having received the COVID-19 vaccine (75.7%) and having been tested for COVID-19 (74.7%). Of those who had tested for COVID-19, 29.1% reported a positive test result. See Table 2 for participant background characteristics.

#### 3.2.1. Trusted Sources of Information

The most trusted sources of COVID-19 information were health care providers (*M* = 3.61, *SD* = 0.72), the Coronavirus Task Force (*M* =3.27, *SD* = 0.87), the U.S. government (*M* = 3.04, *SD* = 0.94), close friends and family members (*M* = 2.91, *SD* = 0.96), the news on the radio/tv/online/newspapers (*M* =2.83, *SD* = 0.95), “your” faith leader (*M* = 2.76, *SD* = 1.12), people “you” go to work or class with or other people “you” know (*M* = 2.74, *SD* = 1.02), and “your” contacts on social media (*M* =2.56, *SD* = 1.06). The five most trusted and credible sources of information who were identified by the select all that apply option were doctor or health care provider (67.7%), government website (e.g., CDC, FDA, etc.) (41.8%), the World Health Organization (37.8%), a community health clinic (37.8%), and pharmacists (32.1%).

#### 3.2.2. Trust and Vaccine Uptake

A logistic regression model examined the impact of trusted sources of COVID-19 information on COVID-19 vaccine uptake. Eight independent variables were entered into the model in the order they were assessed in the original survey (see Table 3). The full model containing all predictors was statistically significant, χ^2^ (8, *N* = 389) = 61.81, *p* < 0.001, indicating that the model was able to distinguish between respondents who received and did not receive the COVID-19 vaccine. The model as a whole explained between 14.7% (Cox and Snell R square) and 22.3% (Nagelkerke R squared) of the variance in COVID-19 vaccine uptake and correctly classified 78.1% of cases. As shown in Table 3, only two of the independent variables made a unique statistically significant contribution to the model: (1) doctor or health care provider and (2) people you go to work or class with or other people you know (acquaintances). The odds ratio of 2.56 indicates that for each unit increase in trust in one’s “doctor or health care provider”, participants were 2.56 times more likely to have received the COVID-19 vaccine. In other words, each additional one-unit increase in trust in doctors or health care providers is associated with a 156% increase in the odds of COVID-19 vaccine uptake. The odds ratio of 0.66 indicates that for each unit increase in trust in the “people you go to work or class with or other people you know”, participants were 0.66 times less likely to have received the COVID-19 vaccine. In other words, each additional one-unit increase in trust in acquaintances is associated with a 34% decrease in the odds of COVID-19 vaccine uptake.

#### 3.2.3. Perceptions and Vaccine Uptake

A logistic regression model examined the impact of COVID-19 health perceptions on COVID-19 vaccine uptake. Five independent variables were entered into the model (perceived efficacy of COVID-19 protective behaviors, perceived COVID-19 invulnerability, perceived effectiveness of the COVID-19 vaccine, fear of COVID-19 vaccine side-effects, and personal health importance). The full model containing all predictors was statistically significant, χ^2^ (5, *N* = 475) = 61.81, *p* < 0.001, indicating that the model was able to distinguish between respondents who received and did not receive the COVID-19 vaccine. The model as a whole explained between 23.6% (Cox and Snell R square) and 36.1% (Nagelkerke R squared) of the variance in COVID-19 vaccine uptake and correctly classified 77.9% of cases. As shown in Table 4, three of the independent variables made a unique statistically significant contribution to the model: (1) perceived COVID-19 invulnerability, (2) perceived effectiveness of the COVID-19 vaccine, and (3) fear of COVID-19 vaccine side-effects. The strongest predictor was perceived effectiveness of the COVID-19 vaccine with an odds ratio of 3.50. Specifically, for each unit increase in perceived effectiveness of the COVID-19 vaccine, participants were 3.5 times more likely to have received the COVID-19 vaccine. The odds ratio of 0.57 indicates that for each unit increase in fear of COVID-19 side effects, participants were 0.57 times less likely to have received the COVID-19 vaccine. The odds ratio of 0.51 indicates that for each unit increase in perceived invulnerability, participants were 0.51 times less likely to have received the COVID-19 vaccine.

## 4. Discussion

Several important findings emerged in the current study highlighting the need for utilizing an integrated, multifactorial approach to increase COVID-19 testing and to identify factors associated with COVID-19 vaccine uptake in underserved communities. More than 4000 community members were tested for COVID-19 in our study, and our findings suggest that one out of two community members testing for COVID-19 will test positive. If these COVID-19-positive individuals had not had access to the test, they may have unintentionally spread COVID-19. Many studies have emphasized the importance of implementing programs to increase COVID-19 testing in underserved populations [20,21,22]. In a study examining equitable access to COVID-19 testing and vaccination in underserved communities, the following conditions were reported as important for increasing COVID-19 testing and vaccine uptake in underserved communities: “(1) accessibility and available services; (2) culturally and linguistically competent programming; (3) investment in trusted community and faith leaders; (4) social safety nets to provide ancillary services” [22]. The current study addressed all four of these conditions in our underserved community by increasing access to COVID-19 testing among 4000 community members and using trusted CHWs to navigate individuals who do not speak English and/or are from lower SES to testing sites.

Findings from our community survey indicate that trust is a primary factor associated with vaccine uptake. The most trusted sources of COVID-19 information were healthcare providers, the Coronavirus Task Force, and the U.S. government. Moreover, we found that the odds of COVID-19 vaccine uptake increased by over 100% in individuals who trust healthcare providers. Recent studies are beginning to explore the role of trust and its impact on COVID-19 preventive behaviors, including vaccination [23,24,25,26,27]. The latter studies reveal similar patterns that suggest that trusting the government is associated with engagement in COVID-19 preventive behaviors. Despite the scarce literature examining the impact of trust sources of information on COVID-19 preventive behaviors, there is a plethora of research investigating the impact of trusted sources of information on human papillomavirus (HPV) vaccine acceptance. In a study examining parental beliefs about cancer, the odds of accepting the HPV vaccine were higher in individuals who trusted doctors or health professionals (*OR* = 1.30, *p* = 0.001) and government agencies (*OR* = 1.29, *p* < 0.001) [27]. Moreover, a literature review examining the role of trust on HPV vaccine uptake in racial and ethnic minorities reported that HPV vaccine uptake was lower in communities of color who mistrust government agencies, healthcare providers, or pharmaceutical companies [28]. A study examining HPV vaccine acceptance in Hispanic males reported that recommendations from a healthcare provider predicted HPV vaccine acceptance (*β* = 0.11, *t* = 2.01, *p* = 0.048) [29]. Improvement in the trust that individuals have towards healthcare providers, government agencies, and pharmaceutical companies seems crucial for increasing vaccinations. In the current study, 67.7% of the sample trusted healthcare providers; however, less than half (41.8%) of our sample reported trust in government websites (e.g., CDC, FDA, etc.). Future studies should explore the rational for trusting and mistrusting various sources of information to inform the development of campaigns utilizing trusted sources of information (e.g., healthcare providers) for recommending vaccines.

Lastly, our study revealed that COVID-19 health perceptions are critical predictors of COVID-19 vaccine uptake. Perceived COVID-19 invulnerability, perceived effectiveness of the COVID-19 vaccine, and fear of COVID-19 vaccine side-effects emerged as statistically significant predictors of COVID-19 vaccine uptake. These findings are consistent with another study which reported that perceived susceptibility of being infected by COVID-19 and perceived benefits of vaccination predict vaccine acceptance [30]. In the latter study, the most common reason for COVID-19 vaccine hesitancy was fear of future vaccine side effects [30]. Notably, behavioral frameworks such as the health belief model (HBM) are useful for understanding behaviors such as accepting a vaccine [31]. The HBM proposes that several factors can predict whether one engages in a behavior including perceived susceptibility (i.e., belief that one is susceptible to being infected with COVID-19), perceived benefits (i.e., belief that the COVID-19 vaccine will prevent serious infection), and perceived barriers (i.e., belief that the COVID-19 vaccine does not cause adverse reactions).

## 5. Conclusions

Although the SARS-CoV-2 pandemic upended the world and laid bare the inequities that underserved populations experience, particularly communities of color including Hispanics, the BBRC was able to utilize core facilities and mobilize BBRC faculty to address the needs of the community. The deployment of CHWs was crucial for navigating community members who were not fluent in English and/or of lower SES to testing sites. The community survey results provided data highlighting the important role of trusted healthcare providers to recommend COVID-19 vaccines. Additionally, our findings support that behavioral frameworks such as the HBM are useful for predicting COVID-19 vaccine uptake. Combined, these findings have implications for the development of public health programs aimed at increasing COVID-19 testing and vaccinations in underserved populations.

## Figures and Tables

**Table 1:** Demographic characteristics (*N* = 502).

Variable	Total Responses	Percentage of Responses
Sex assigned at birth		
Male	234	46.6%
Female	263	52.4%
Intersex	1	0.2%
Missing	4	0.8%
Ethnicity		
Non-Hispanic	146	29.1%
Hispanic	335	66.7%
Prefer not to answer	15	3.0%
Missing	6	1.2%
Race ^†^		
American Indian or Alaskan Native	38	7.6%
African American	73	14.5%
Asian	6	1.2%
Native Hawaiian or Pacific Islander	12	2.4%
White	342	68.1%
Other	18	3.6%
Prefer not to answer	20	4%
Education		
5th grade or less	1	0.2%
6th to 8th grade	6	1.2%
9th to 12th grade, no diploma	12	2.4%
High school graduate or GED completed	48	9.6%
Some college level/Technical/Vocational	147	29.3%
Bachelor’s degree	222	44.2%
Other advanced degree (MA, PhD, MD)	59	11.8%
Prefer not to answer	5	1.0%
Missing	2	0.4%
Household income in 2019 before taxes		
Less than $15,000	31	6.2%
$15,000–$19,999	23	4.6%
$20,000–$24,999	46	9.2%
$25,000–$34,999	64	12.7%
$35,000–$49,999	88	17.5%
$50,000–$74,999	84	16.7%
$75,000–$99,999	86	17.1%
$100,000 and above	60	12%
Prefer not to answer	20	4%

Note: ^†^ Item is a “select all that apply” response and therefore does not total up to 100%.

**Table 2:** Background information (*N* = 502).

Variable	Total Responses	Percentage of Responses
Health insurance or health care plan		
I do not have health insurance	95	18.9%
Private	206	41%
Public (Medicare, Medicaid, Tricare)	176	35.1%
Don’t know	8	1.6%
Prefer not to answer	14	2.8%
Missing	3	0.6%
Did you lose health coverage because of the COVID-19 pandemic?		
No	372	74.1%
Yes	112	22.3%
Prefer not to answer	14	2.8%
Missing	4	0.8%
Received COVID-19 Vaccine		
No	114	22.7%
Yes	380	75.7%
Prefer not to answer	5	1.0%
Don’t know	2	0.4%
Missing	1	0.2%
Ever Tested for COVID-19		
No	121	24.1%
Yes	375	74.7%
Prefer not to answer	3	0.6%
Missing	3	0.6%
Ever Tested Positive for COVID-19		
No	222	44.2%
Yes	146	29.1%
Prefer not to answer	3	0.6%
Missing	131	26.1%

**Table 3:** Logistic regression predicting COVID-19 vaccine uptake from trusted sources.

	B	S.E.	Wald	df	*p*	Odds Ratio	95% C.I. for Odds Ratio	
							Lower	Upper
1. Your faith leader	−0.20	0.16	1.60	1	0.206	0.82	0.60	1.12
2. Your doctor or health care provider	0.94	0.18	26.84	1	<0.001	2.56	1.79	3.65
3. Your close friends and members of your family	−0.24	0.18	1.79	1	0.181	0.79	0.56	1.12
4. People you go to work or class with or other people you know	−0.41	0.20	4.37	1	0.036	0.66	0.45	0.97
5. News on the radio, TV, online, or in newspapers	0.09	0.18	0.26	1	0.613	1.10	0.77	1.58
6. Your contacts on social media	−0.04	0.18	0.04	1	0.847	0.97	0.68	1.39
7. The U.S. government	0.04	0.20	0.04	1	0.839	1.04	0.70	1.55
8. The U.S. Coronavirus Task Force	0.30	0.19	2.44	1	0.119	1.35	0.93	1.97
Constant	−0.85	0.82	1.08	1	0.298	0.43		

**Table 4:** Logistic regression predicting COVID-19 vaccine uptake from COVID-19 health perceptions.

	B	S.E.	Wald	df	*p*	Odds Ratio	95% C.I. for Odds Ratio	
							Lower	Upper
1. Perceived efficacy of COVID-19 protective behaviors	0.22	0.17	1.71	1	0.191	1.25	0.89	1.75
2. Perceived COVID-19 invulnerability	−0.68	0.18	13.98	1	<0.001	0.51	0.36	0.73
3. Perceived effectiveness of the COVID-19 vaccine	1.25	0.18	48.62	1	<0.001	3.50	2.46	4.97
4. Fear of COVID-19 vaccine side-effects	−0.57	0.18	10.66	1	0.001	0.57	0.40	0.80
5. Personal health importance	0.35	0.20	2.99	1	0.083	1.42	0.96	2.11
Constant	−1.45	0.95	2.30	1	0.129	0.24		

## Data Availability

The data presented in this study are available on request from the corresponding author. The data are not publicly available due to a repository currently being created to host all data from the projects funded by the same agency.
